# Treatment of critically ill patients with cefiderocol for infections caused by multidrug-resistant pathogens: review of the evidence

**DOI:** 10.1186/s13613-023-01146-5

**Published:** 2023-06-15

**Authors:** Pierluigi Viale, Christian E. Sandrock, Paula Ramirez, Gian Maria Rossolini, Thomas P. Lodise

**Affiliations:** 1 Infectious Disease Unit, IRCCS Policlinico di Sant’Orsola, Bologna, Italy; 2 Department of Medical and Surgical Science, Alma Mater Studiorum-Università di Bologna, Bologna, Italy; 3 Division of Pulmonary, Critical Care and Sleep Medicine, Department of Internal Medicine, University of California, Davis, Sacramento, CA USA; 4 Servicio de Medicina Intensiva, Hospital Universitario y Politécnico la Fe, Valencia, Spain; 5 Department of Experimental and Clinical Medicine, University of Florence, Florence, Italy; 6 Microbiology and Virology Unit, Careggi University Hospital, Florence, Italy; 7 Department of Pharmacy Practice, Albany College of Pharmacy and Health Sciences, Albany, NY USA

**Keywords:** Appropriate antibiotic, Cefiderocol, Critically ill, Dosing, Multidrug-resistant Gram-negative bacteria, Nosocomial pneumonia, Sepsis

## Abstract

Appropriate antibiotic treatment for critically ill patients with serious Gram-negative infections in the intensive care unit is crucial to minimize morbidity and mortality. Several new antibiotics have shown in vitro activity against carbapenem-resistant Enterobacterales (CRE) and difficult-to-treat resistant *Pseudomonas aeruginosa*. Cefiderocol is the first approved siderophore beta-lactam antibiotic with potent activity against multidrug-resistant, carbapenem-resistant, difficult-to-treat or extensively drug-resistant Gram-negative pathogens, which have limited treatment options. The spectrum of activity of cefiderocol includes drug-resistant strains of *Acinetobacter baumannii*, *P. aeruginosa*, *Stenotrophomonas maltophilia*, *Achromobacter* spp. and *Burkholderia* spp. and CRE that produce serine- and/or metallo-carbapenemases. Phase 1 studies established that cefiderocol achieves adequate concentration in the epithelial lining fluid in the lung and requires dosing adjustment for renal function, including patients with augmented renal clearance and continuous renal-replacement therapy (CRRT); no clinically significant drug–drug interactions are expected. The non-inferiority of cefiderocol versus high-dose, extended-infusion meropenem in all-cause mortality (ACM) rates at day 14 was demonstrated in the randomized, double-blind APEKS–NP Phase 3 clinical study in patients with nosocomial pneumonia caused by suspected or confirmed Gram-negative bacteria. Furthermore, the efficacy of cefiderocol was investigated in the randomized, open-label, pathogen-focused, descriptive CREDIBLE–CR Phase 3 clinical study in its target patient population with serious carbapenem-resistant Gram-negative infections, including hospitalized patients with nosocomial pneumonia, bloodstream infection/sepsis, or complicated urinary tract infections. However, a numerically greater ACM rate with cefiderocol compared with BAT led to the inclusion of a warning in US and European prescribing information. Cefiderocol susceptibility results obtained with commercial tests should be carefully evaluated due to current issues regarding their accuracy and reliability. Since its approval, real-world evidence in patients with multidrug-resistant and carbapenem-resistant Gram-negative bacterial infections suggests that cefiderocol can be efficacious in certain critically ill patient groups, such as those requiring mechanical ventilation for COVID-19 pneumonia with subsequently acquired Gram-negative bacterial superinfection, and patients with CRRT and/or extracorporeal membrane oxygenation. In this article, we review the microbiological spectrum, pharmacokinetics/pharmacodynamics, efficacy and safety profiles and real-world evidence for cefiderocol, and look at future considerations for its role in the treatment of critically ill patients with challenging Gram-negative bacterial infections.

**Supplementary Information:**

The online version contains supplementary material available at 10.1186/s13613-023-01146-5.

## Introduction

Healthcare-associated infections, including hospital-acquired pneumonia (HAP), ventilator-associated pneumonia (VAP), bloodstream infection (BSI), and catheter-associated complicated urinary tract infections (CA-UTI) are leading causes of morbidity and mortality in critically ill patients [1–3]. A driving factor in poor outcomes is a delay in the administration of appropriate antibiotics, particularly in patients with serious multidrug-resistant (MDR) infections [4–7], underlining the importance of recognizing risk factors for MDR pathogens [8]. In critically ill patients, the most concerning MDR pathogens are: carbapenem-resistant (CR) and difficult-to-treat resistant (DTR, i.e., not susceptible to all high-efficacy and low-toxicity antibiotics [4]) *Pseudomonas aeruginosa*; CR *Acinetobacter baumannii*; extended-spectrum beta-lactamase (ESBL)-producing and CR Enterobacterales (CRE); and *Stenotrophomonas maltophilia* [9–15].

In recent years, several new agents based on combinations of old beta-lactams and new beta-lactamase inhibitors with activity against highly resistant Gram-negative pathogens have been approved (e.g., ceftazidime–avibactam, meropenem–vaborbactam, imipenem–relebactam) [13]. Against this backdrop, we look at the role of the first siderophore cephalosporin antibiotic to be approved, cefiderocol. Developed specifically to target CR Gram-negative bacterial infections [16, 17], cefiderocol binds iron, an essential nutrient for bacterial growth, and enters bacterial cells not only by passive diffusion through porin channels but also through iron transport channels [16, 17]. Cefiderocol is mostly stable against a broad range of serine- and metallo-beta-lactamases (MBLs), although it can be weakly hydrolyzed by certain mutant isoforms of these enzymes [18–21]. Unlike for carbapenems and other standard-of-care beta-lactam antibiotics, porin loss and efflux pump upregulation mechanisms of antimicrobial resistance in Gram-negative bacteria have limited effect on the overall in vitro activity of cefiderocol [19, 22].

This review summarizes the published literature on the in vitro susceptibility of cefiderocol against highly resistant Gram-negative bacterial isolates across surveillance programs, mechanisms of resistance, pharmacokinetics (PK), PK/pharmacodynamics (PK/PD) in preclinical models of infections, and clinical data. It also discusses the characteristics of cefiderocol relative to recently approved beta-lactam–beta-lactamase inhibitor (BL–BLI) combinations, and covers overlapping and differential potential roles of cefiderocol and BL–BLIs in therapy within the current antibiotic landscape. Finally, consideration is given to future studies that would help to better define the role of cefiderocol relative to recently approved BL–BLIs in the treatment of patients with highly resistant infections.

## Microbiology and spectrum of activity of cefiderocol

Cefiderocol has a broad spectrum of activity against aerobic Gram-negative bacteria frequently found in the intensive care unit (ICU), including MDR and CR strains of Enterobacterales, *P. aeruginosa, A.* *baumannii*, and *S. maltophilia*, the latter of which is intrinsically resistant to carbapenems [19, 23]. Cefiderocol has no in vitro activity against Gram-positive bacteria and anaerobes [19]. Clinical breakpoints for interpretation of the results of cefiderocol susceptibility testing are available from Clinical and Laboratory Standards Institute (CLSI), the US Food and Drug Administration (FDA) and the European Committee on Antimicrobial Susceptibility Testing (EUCAST) (Table 1) [24–26]. The interpretive criteria are not identical, which may result in discrepancies in categorization of isolates of the same species exhibiting the same cefiderocol minimum inhibitory concentration (MIC) values, depending on the breakpoint system adopted.

**Table 1:** Cefiderocol clinical breakpoints for interpretation of in vitro susceptibility testing results by Clinical and Laboratory Standards Institute (CLSI) [24], US Food and Drug Administration (FDA) [25], and European Committee on Antimicrobial Susceptibility Testing (EUCAST) [26] for Gram-negative pathogens

	Interpretive criteria according to Minimum inhibitory concentration (µg/mL)		
	S^a^	I^b^	R^c^
*Enterobacterales*			
CLSI	≤ 4	8	≥ 16
FDA	≤ 4	8	≥ 16
EUCAST	≤ 2	–	> 2
*Pseudomonas aeruginosa*			
CLSI	≤ 4	8	≥ 16
FDA	≤ 1	2	≥ 4
EUCAST	≤ 2	–	> 2
*Acinetobacter baumannii*			
CLSI	≤ 4	8	≥ 16
FDA	≤ 1	2	≥ 4
EUCAST^d^	IE	–	IE
*Stenotrophomonas maltophilia*			
CLSI^e^	≤ 1	–	–
FDA^f^	–	–	–
EUCAST^d^	IE	–	IE

^a^S: susceptible for CLSI and FDA; susceptible, standard dosing regimen for EUCAST.

^b^I: intermediate for CLSI and FDA; susceptible, increased exposure for EUCAST; EUCAST does not foresee an "I" category for cefiderocol for Enterobacterales or *P. aeruginosa*.

^c^R: resistant.

^d^EUCAST has not set clinical breakpoints for cefiderocol for *Acinetobacter and S. maltophilia* due to insufficient evidence (IE).

^e^CLSI does not foresee “I” and “R” categories for S. *maltophilia*.

^f^FDA does not foresee clinical breakpoints for S. *maltophilia*.

The in vitro activity of cefiderocol was investigated in large surveillance studies and in smaller independent investigations. Among 800 Gram-negative clinical isolates derived from ICU patients with infections of the lower respiratory tract (LRTI) and urinary tract (UTI), or with wound infection or BSI, cefiderocol MICs were ≤ 4 μg/mL against all isolates, including carbapenem–non-susceptible Enterobacterales, *P. aeruginosa*, and *A. baumannii* [27].

The 5-year multinational SIDERO surveillance program, based on CLSI breakpoints for all species, showed that 96.7% of CREs, 99.8% of CR strains of *P. aeruginosa*, 94.2% of CR strains of *A. baumannii*, and 98.6% of *S. maltophilia* were susceptible to cefiderocol [28]. *Burkholderia* spp. do not have CLSI susceptibility breakpoints; however, cefiderocol MIC_90_ was 0.5 μg/mL, suggesting low MIC values for most isolates [28]. For carbapenem–non-susceptible *Achromobacter* spp., the cefiderocol MIC_90_ was 4 μg/mL in a global collection of isolates [29].

High in vitro activity of cefiderocol against Enterobacterales, *P. aeruginosa*, *A. baumannii*, and *S. maltophilia* was also observed in the 2020 SENTRY multinational surveillance program. In addition, cefiderocol was the most potent antibiotic against the different resistance phenotypes (Table 2) [30]. Overall, 89.2–95.9% of Enterobacterales isolates resistant to imipenem–relebactam, meropenem–vaborbactam, or ceftazidime–avibactam were susceptible to cefiderocol according to CLSI criteria and 54.1–70.7% of the same isolates were susceptible to cefiderocol based on EUCAST criteria (Table 2) [30], reflecting the different breakpoints established by the two committees. Among *P. aeruginosa* isolates resistant to ceftolozane–tazobactam, imipenem–relebactam, or ceftazidime–avibactam, susceptibility rates of 88.3%, 100%, and 91.6% of isolates, respectively, were reported for cefiderocol by CLSI criteria and of 85%, 100%, and 89.2% of isolates by EUCAST criteria, respectively (Table 2) [30].

**Table 2:** Comparative susceptibility to cefiderocol versus ceftazidime–avibactam, ceftolozane–tazobactam, imipenem–relebactam, meropenem–vaborbactam, colistin of Gram-negative bacterial species

	Cefiderocol			Ceftazidime–avibactam			Ceftolozane–tazobactam			Imipenem–relebactam			Meropenem–vaborbactam			Colistin^a^			Refs
	MIC_90_ (µg/mL)	Susceptibility rate (%)		MIC_90_ (µg/mL)	Susceptibility rate (%)		MIC_90_ (µg/mL)	Susceptibility rate (%)		MIC_90_ (µg/mL)	Susceptibility rate (%)		MIC_90_ (µg/mL)	Susceptibility rate (%)		MIC_90_ (µg/mL)	Susceptibility rate (%)		
		CLSI	EUCAST		CLSI	EUCAST		CLSI	EUCAST		CLSI	EUCAST		CLSI	EUCAST		CLSI	EUCAST	
Enterobacterales	1	99.8	98.3	0.5	99.2	NR	2	91.7	NR	NT	NT	NT	NT	NT	NT	1	96.8	NR	[28]
	0.5	99.8	99.1	0.25	99.5	99.5	NT	NT	NT	0.5	94.8	98.9^b^	0.06	99.4	99.5	> 8	83.6	83.6	[30]
CRE	4	96.7	79.9	> 64	77.0	NR	> 64	7.8	NR	NT	NT	NT	NT	NT	NT	> 8	80.5	NR	[28]
	4	98.2	87.6	> 32	81.7	81.7	NT	NT	NT	> 8	63.9	71.0^b^	> 8	71.0	75.7	> 8	78.7	78.7	[30]
M/V-R Enterobacterales	4	95.1	70.7	> 32	43.9	43.9	NT	NT	NT	> 8	2.4	7.3^b^	> 8	0	0	> 8	48.8	48.8	[30]
I/R-R Enterobacterales	4	95.9	69.4	> 32	40.8	40.8	NT	NT	NT	> 8	0	0^b^	> 8	16.3	24.5	> 8	55.1	55.1	[30]
CZA-R Enterobacterales	8	89.2	54.1	> 32	0	0	NT	NT	NT	> 8	5.4	8.1^b^	> 8	29.7	37.8	> 8	56.8	56.8	[30]
*P. aeruginosa*	0.5	99.9	99.4	8	93.8	NR	2	94.0	NR	NT	NT	NT	NT	NT	NT	2	99.3	NR	[28]
	0.5	99.6	99.4	4	96.4	96.4	2	96.1	96.1	1	96.4	96.4	NT	NT	NT	1	99.6	99.6	[30]
CR *P. aeruginosa*	1	99.8	98.5	32	75.0	NR	> 64	76.1	NR	NT	NT	NT	NT	NT	NT	1	98.5	NR	[28]
XDR *P. aeruginosa*	1	97.3	96.9	32	73.4	73.4	> 16	72.3	72.3	> 8	73.0	73.0	NT	NT	NT	1	99.2	99.2	[30]
XDR *P. aeruginosa*	4	97.4	NA	> 16	57.0	NA	32	66.8	NA	16	49.8	NA	NT	NT	NT	NT	NT	NT	[128]
C/T-R *P. aeruginosa*	8	88.3	85.0	> 32	25.0	25.0	> 16	0	0	> 8	43.3	43.3	NT	NT	NT	1	100	100	[30]
I/R-R *P. aeruginosa*	1	100	100	> 32	35.4	35.4	> 16	20.8	20.8	> 8	0	0	NT	NT	NT	1	100	100	[30]
CZA-R *P. aeruginosa*	4	91.6	89.2	> 32	0	0	> 16	37.3	37.3	> 8	47.0	47.0	NT	NT	NT	1	100	100	[30]
*A. baumannii*	1	96.0	94.1	> 64	NA	NA	> 64	NA	NA	NT	NT	NT	NT	NT	NT	2	92.7	NR	[28]
	1	97.7	95.7^c^	NT	NT	NT	NT	NT	NT	> 8	53.1	53.1	NT	NT	NT	8	86.3	86.3	[30]
CR *A. baumannii*	2	94.2	91.1	> 64	NA	NA	> 64	NA	NA	NT	NT	NT	NT	NT	NT	> 8	87.2	NR	[28]
	2	95.8	91.5^c^	NT	NT	NT	NT	NT	NT	> 8	0.3	0.3	NT	NT	NT	> 8	76.4	76.4	[30]
*S. maltophilia*	0.25	98.6	99.6	64	NA	NA	> 64	NA	NA	NT	NT	NT	NT	NT	NT	> 8	NA	NA	[28]
	0.5	97.9	99.7^c^	NT	NT	NT	NT	NT	NT	NT	NT	NT	NT	NT	NT	> 8	NA	NA	[30]
*Achromobacter* spp.	0.5	NA	NA	16	NA	NA	NT	NT	NT	2	NA	NA	4	NA	NA	4	NA	NA	[29]
CR *Achromobacter* spp.	4	NA	NA	> 16	NA	NA	NT	NT	NT	> 16	NA	NA	> 16	NA	NA	8	NA	NA	[29]
*Burkholderia* spp.	0.5	NA	NA	8	NA	NA	32	NA	NA	NT	NT	NT	NT	NT	NT	> 8	NA	NA	[28]
	0.5	NA	NA	8	NA	NA	32	NA	NA	4	NA	NA	4	NA	NA	> 8	NA	NA	[147]
CR *Burkholderia* spp.	1	NA	NA	16	NA	NA	> 64	NA	NA	4	NA	NA	4	NA	NA	> 8	NA	NA	[147]

Adapted from [28–30, 128, 147]

*CFDC* cefiderocol*, CLSI* Clinical and Laboratory Standards Institute, *COL* colistin*, CR* carbapenem resistant, *CRE* carbapenem-resistant Enterobacterales, *CZA* ceftazidime–avibactam*, C/T* ceftolozane–tazobactam*, I/R* imipenem–relebactam*, MIC* minimum inhibitory concentration, *M/V* meropenem–vaborbactam, *NA* susceptibility breakpoint not available, *NR* not reported, *NT* not tested, *R* resistant, *XDR* extensively drug resistant

^a^Only intermediate category is applicable by CLSI

^b^CLSI/FDA breakpoints were applied to all species but were approved for Enterobacterales except *Morganella*, *Proteus*, and *Providencia* and EUCAST excludes Morganellaceae

^c^EUCAST non-species-specific PK/PD breakpoint used

## Cefiderocol susceptibility testing issues

The mechanism of cefiderocol entry into the bacterial cell requires expression of specific siderophore (iron transport) uptake systems under low iron concentrations encountered in infected tissues in vivo, which accounts for the remarkable spectrum of antimicrobial activity. This implies that the reference broth microdilution (BMD) for cefiderocol susceptibility testing must use iron-depleted medium to avoid misleading results (i.e., false resistance) [31–33]. While this prevented an easy addition of cefiderocol to panels used by several antimicrobial susceptibility testing (AST) platforms, some commercial systems for cefiderocol MIC testing have been developed. However, these systems were shown to be affected by accuracy, reproducibility, and bias issues [34–37]. According to EUCAST, disk diffusion can be used as a starting approach to cefiderocol testing by diagnostic laboratories, but results falling into the area of technical uncertainty (ATU) should either be confirmed by the reference BMD with iron-depleted, cation-adjusted Mueller–Hinton medium or cautionary considered indicative of resistance [37, 38], leading to an overall overestimation of resistance. In fact, it was shown that more than 20% of isolates of MDR Gram-negatives may yield cefiderocol results falling into the ATU when tested by disk diffusion [35]. Moreover, disk diffusion can also be affected by remarkable variability depending on the various combinations of disks and agar media manufacturers [34, 37]. Additional commercial systems for cefiderocol susceptibility testing have recently been developed, and evaluations of the available systems are underway with variable results [39–43].

## Pharmacokinetics in healthy subjects and critically ill patients

In two Phase 1 studies in healthy subjects, single and multiple intravenous doses of cefiderocol up to 4000 mg were well-tolerated and exhibited a linear PK profile [44, 45]. Dose-proportional increases in maximum serum concentration (*C*_max_) and area under the concentration–time curve (AUC_0-∞_) were observed between 100 to 4000 mg [44, 45]. The mean plasma half-life of cefiderocol was 1.98–2.74 h and the PK of cefiderocol did not change with multiple dosing in healthy subjects [44]. Protein binding was estimated at approximately 58% in healthy subjects and was not significantly altered with renal impairment [46, 47]. Cefiderocol (1 g, 1-h infusion, radio-labeled) was primarily excreted unchanged in the urine, with metabolic pathways providing only minor excretion routes [48]. Following administration of cefiderocol 2 g, in 1-h infusion, the AUC (212.0 to 872.5 µg • h/mL) and half-life (2.8 to 9.6 h) increased and renal clearance decreased (4.7 to 1.1 L/h) in patients with mild, moderate or severe renal impairment (including those patients with or without hemodialysis) compared with subjects with normal renal function [47], suggesting that cefiderocol dose adjustment is needed with renal function. In vitro data demonstrating no interactions of cefiderocol with drug transporter systems have been confirmed in studies using furosemide, metformin, and rosuvastatin in healthy volunteers [49, 50]. In vitro data indicate that cefiderocol may induce CYP3A4 in the liver; thus, patients should be monitored for potential reduced efficacy when substrates of CYP3A4 enzyme are co-administered with cefiderocol [51]. Studies on epithelial lining fluid (ELF) penetration of cefiderocol confirmed its effective tissue penetration into the lung. In healthy volunteers, a single 2 g 1-h infusion resulted in ~ 24% ELF to plasma ratio of free cefiderocol [46].

Data are also available on the PK of cefiderocol in plasma and ELF in critically ill patients. In a small French study in seven ICU patients, mainly with VAP (six patients), the PK profile of cefiderocol at steady state was investigated after multiple dosing of 2 g, 3-h infusions [52]. The mean (± standard deviation [SD]) minimum serum concentration (*C*_min_) of cefiderocol prior to the start of infusion (*C*_0_) was 42.1 ± 20.1 mg/L, the mean *C*_max_ was 77.1 ± 12.9 mg/L, the mean AUC_0–8 h_ was 448 ± 155 mg • h/L and the half-life was 13 ± 11.3 h [52]. In one patient with augmented renal clearance (ARC), the half-life of cefiderocol was lower (1.4 h) than in the rest of the ICU population [52]. The PK of cefiderocol in critically ill patients has also been reported for five patients with septic shock due to MDR Gram-negative bacteria, all of whom had varying degrees of acute renal injury; three required continuous renal replacement therapy (CRRT), one required an additional cytokine adsorber, and two patients also required extracorporeal membrane oxygenation (ECMO) [53]. With a target of 100% for the percentage of time during the dosing period that the free drug concentration in the plasma exceeds the MIC (%*f*T_>1×MIC_; overall *C*_min_ > 4 μg/mL), none of the initial or renally adapted dosing regimens were suboptimal. *C*_min_ levels ranged between 25 and 70 μg/mL; however, there was a reduction in *C*_min_ in a patient receiving cytokine adsorber therapy [53]. Cefiderocol half-life was prolonged for at least 8 h in all patients [53]. In a Phase 1b study in hospitalized patients with pneumonia under mechanical ventilation, the ELF penetration at steady state increased to ~ 54% after multiple dosing of cefiderocol 2 g in 3-h infusions [54].

The PK of cefiderocol have been further investigated to support dosing under CRRT and ECMO. A recent ex vivo investigation suggested that the effluent rate was the significant parameter determining the clearance of cefiderocol during CRRT. Based on these findings, dosing recommendations according to the effluent rate were incorporated into the cefiderocol prescribing information [49, 55, 56]. In an ex vivo investigation that compared the PK of cefiderocol relative to baseline conditions, cefiderocol concentrations in blood samples collected post-oxygenation were reduced in a similar trend between closed-loop ECMO circuits and the control chambers without an oxygenator [57]. In another study, samples collected from the ECMO circuit on day 9 suggested < 5% loss of cefiderocol, and plasma and lung trough concentrations also remained above the MIC of *P. aeruginosa* [58]. Collectively, these data suggest that there was no significant cefiderocol adsorption to the oxygenator membrane in the ECMO circuit and cefiderocol PK is not expected to be altered in patients with ECMO support [57].

## Preclinical PK/PD investigations

Dose fractionation in vivo preclinical studies in neutropenic murine thigh and lung infection models demonstrated that the PD parameter most closely correlated with the efficacy of cefiderocol was the time the free concentration remained above the MIC (%*f*T_>MIC_) [59]. Based on the efficacy variable of 1-log reduction in bacterial growth, the PD target required to achieve in vivo efficacy differed by bacterial species (i.e., Enterobacterales 64% and 73% [lung, thigh], *P. aeruginosa* 70% and 72% [lung, thigh], *A.* *baumannii* 88% [lung], and *S. maltophilia* 54% [lung]) [59]. In another series of experiments investigating cefiderocol doses up to 250 mg/kg against *P. aeruginosa* in the neutropenic murine thigh infection model, in which 32% protein binding was applied to correct for free drug exposure, ~ 76%, 82%, or 88% T_>MIC_ was required to achieve in vivo bacteriostatic effects, 1-log_10_, or 2-log_10_ killing, respectively [60].

The neutropenic murine thigh infection model was also used to investigate the potential susceptibility breakpoint for cefiderocol at humanized dosing (replicating exposures of 2 g, 3-h infusion every 8 h in humans) against various Gram-negative bacterial species with cefiderocol MICs of 0.12 to > 256 μg/mL [61]. Over 24 h, among isolates with cefiderocol MIC values up to 4 μg/mL, cefiderocol led to bacterial stasis or ≥ 1-log reduction in colony-forming unit (CFU) in 77% of Enterobacterales, 88% of *A. baumannii*, and 85% of *P. aeruginosa* isolates [61]. The suppression of bacterial growth (from stasis to cidal effects [i.e., ≥ 2-log_10_ reduction in CFU]) was sustained for up to 72 h against a total of four meropenem-susceptible and six meropenem-resistant strains of Enterobacterales, *A. baumannii*, and *P. aeruginosa* with cefiderocol MIC values up to 4 μg/mL in another neutropenic murine thigh infection model study [62]. Additional studies in the same model confirmed that sustained in vivo efficacy (up to 72 h) with cefiderocol can be obtained against CR *A. baumannii* without the emergence of in vivo resistance [63]. Humanized dosing of cefiderocol in this model was also efficacious against *P. aeruginosa* isolates with cefiderocol MIC values of 0.063–0.5 μg/mL, with a ≥ 2-log_10_ reduction in CFU at 24 h compared with vehicle-treated controls against all except one isolate [64]. Furthermore, the infusion duration selected for cefiderocol in Phase 3 trials was based on results from an immunocompetent rat respiratory tract infection model, which showed that a 3-h infusion of cefiderocol at humanized dosing showed enhanced efficacy against some isolates of *A. baumannii* and *K. pneumoniae* compared with a 1-h infusion of cefiderocol [65].

Synergism between cefiderocol and avibactam, amikacin, meropenem, and ampicillin–sulbactam at concentrations replicating exposures have been observed in an in vitro chemostat infection model against *A. baumannii* strains that were resistant to cefiderocol [66–68]. In a different in vitro study in time-kill curve experiments, against *A. baumannii* strains with cefiderocol MICs of 16–32 µg/mL, the combination of cefiderocol with generic drugs sulbactam, amikacin, and minocycline also showed synergism in suppression of bacterial growth [68]. In a recent investigation in the neutropenic murine thigh infection model, cefiderocol at humanized dosing in combination with meropenem, ceftazidime–avibactam, or ampicillin–sulbactam was more efficacious against cefiderocol-susceptible *A. baumannii* isolates (MIC = 2 µg/mL) relative to cefiderocol alone [69].

## Dosing and population PK analyses

Based on the preclinical investigations, the standard dosing and infusion time of cefiderocol in patients with normal renal function was modeled as a 2 g, 3-h infusion every 8 h (q8h), which provided > 90% probability of target attainment (PTA) for Gram-negative bacteria with cefiderocol MIC values up to 4 µg/mL [70, 71]. Cefiderocol dosing for patients with renal impairment, initially modeled using PTAs for 75% *f*T_>MIC_, are as follows (all as a 3-h intravenous infusion): mild impairment (creatinine clearance [CrCl] estimated using Cockcroft–Gault equation in the final model as 60 to < 90 mL/min), 2 g q8h; moderate impairment (30 to < 60 mL/min), 1.5 g q8h; severe impairment (15 to < 30 mL/min), 1 g q8h; and end-stage renal disease (< 15 mL/min), 0.75 g q12h [70, 71]. Modeling suggests that a dose of 2 g q6h can provide the same PTA for patients with ARC (CrCl > 120 mL/min) as a dose of 2 g q8h in critically ill patients with normal renal function (Table 3) [71].

**Table 3:** Cefiderocol dosing recommendations

	Dosing recommendation
*Creatinine clearance, mL/min*^*a*^	
≥ 120	2 g, every 6 h, infused over 3 h
90–120	2 g, every 8 h, infused over 3 h
60–90	2 g, every 8 h, infused over 3 h
30–59	1.5 g, every 8 h, infused over 3 h
15–29	1 g, every 8 h, infused over 3 h
< 15 (with or without intermittent hemodialysis^b^)	0.75 g, every 12 h, infused over 3 h
*CRRT*^*c*^*—effluent flow rate*^*d*^*, L/h*	
≥ 4.1	2 g, every 8 h, infused over 3 h
3.1–4	1.5 g, every 8 h, infused over 3 h
2.1–3	2 g, every 12 h, infused over 3 h
≤ 2	1.5 g, every 12 h, infused over 3 h

Adapted from [49]

^a^Creatinine clearance estimated by Cockcroft–Gault formula

^b^Cefiderocol is removed by hemodialysis; cefiderocol dose should be administered immediately after hemodialysis for patients who require intermittent hemodialysis

^c^Includes patients receiving continuous venovenous hemofiltration, continuous venovenous hemodialysis, continuous venovenous hemodiafiltration

^d^Ultrafiltrate flow rate for continuous venovenous hemofiltration, dialysis flow rate for continuous venovenous hemodialysis, ultrafiltrate flow rate plus dialysis flow rate for continuous venovenous hemodiafiltration

*CRRT* continuous renal replacement therapy

The abovementioned cefiderocol dosing regimens were utilized in critically ill patients with nosocomial pneumonia (NP), BSI, and cUTI in the randomized Phase 3 APEKS–NP and CREDIBLE–CR studies, the results of which studies are discussed below [72, 73]. In these Phase 3 studies, cefiderocol doses for treatment were based on renal function determined at screening and during therapy on days 3–4 [72, 73]. An analysis of 187 cefiderocol-treated patients, 120 of whom were in the ICU and > 20% of whom had ARC, reported no significant difference in C_max_ and daily AUC according to renally adjusted dosing [74]. Geometric means of *f*C_min_ and %*f*T > _MIC≤4 μg/mL_ were > 9 μg/mL and > 93%, respectively, indicating adequate exposure for the treatment of susceptible pathogens based on CLSI breakpoint (i.e., MIC ≤ 4 μg/mL), regardless of renal function, infection site, body size, and disease severity [74].

The PTA for cefiderocol was investigated in a population PK modeling study, developed using data from Phase 1–3 studies [71, 75] and involving 3427 plasma concentrations from 516 subjects (91 patients without infection and 425 patients with infection [i.e., pneumonia, BSI/sepsis, cUTI]) [71]. Cefiderocol plasma concentrations were described by a three-compartment model with a proportional error model for intraindividual variability [71]. The effects of CrCl and infection sites on clearance, body weight on volume of distribution in the central and peripheral compartments, albumin concentration and any infection site on the volume of distribution in the peripheral compartment were included in the final model [71]. CrCl was the most significant covariate on cefiderocol PK. The %*f*T_>MIC_ was 100% in 97% of patients in both Phase 3 studies and the PTA for 100% *f*T_>MIC_ was > 90% with MICs of ≤ 4 mg/mL for most infection sites and renal function groups [71]. No PK/PD correlation was found between plasma exposures and clinical outcomes, microbiological outcomes, or vital status, because the exposure level was very high in most patients [71, 75]. For simulated patients with infections caused by pathogens with cefiderocol MICs of ≤ 4 µg/mL, the PTA for 75% *f*T_>MIC_ was > 95% for all infection sites and renal function groups, while for 100% *f*T_>MIC_, the PTA was > 90% for all infection sites and renal function groups, except for patients with BSI and normal renal function, in whom the PTA was 85% [71].

ELF concentrations were also explored in the population PK analysis of the Phase 3 studies. The estimated %*f*T_>MIC,ELF_ was 100% in 89.3% of patients in CREDIBLE–CR and 97.9% of patients in APEKS–NP [75], supporting the adequacy of cefiderocol ELF concentrations to treat Gram-negative bacterial pneumonia caused by pathogens with a cefiderocol MIC of ≤ 4 mg/L [54, 76]. In Monte Carlo simulations for patients with NP, the PTA in ELF was found as > 99.5% for pathogens with cefiderocol MIC values ≤ 2 µg/mL and > 87% for MIC values of ≤ 4 µg/mL, across all renal function groups [76].

## Compatibility and solubility for intravenous infusion

Cefiderocol has shown good compatibility with frequently used intravenous infusions for hospitalized patients, with a compatibility rate of 69% of 91 medicinal products in a Y-site simulated investigation [77]. Where medications (e.g., dobutamine hydrochloride, lorazepam, methylprednisolone acetate, propofol, rocuronium bromide, tobramycin sulfate, vancomycin hydrochloride) are not compatible with cefiderocol, separate intravenous catheter administration should be used [77]. The MINI-BAG Plus Container System and VIAL-MATE Adaptor (Baxter HealthCare Co., Deerfield, IL, USA) are both compatible with the cefiderocol 1 g vial, facilitating cefiderocol ease of use and minimizing the risk of contamination and environmental exposure [78].

## Clinical experience

### Efficacy

Cefiderocol has demonstrated efficacy in patients with serious infections in one randomized, prospective, Phase 2 (APEKS–cUTI) and two randomized, prospective, active-controlled or parallel-group Phase 3 (APEKS–NP, CREDIBLE–CR) clinical studies in critically ill patients with NP, BSI/sepsis, and cUTI, who were at risk of or were infected by MDR and CR Gram-negative pathogens (Additional file 1: Table S1) [72, 73, 79]. It should be noted that the APEKS–cUTI and APEKS–NP studies excluded patients with pathogens known to be CR at study entry as the comparator agent was a carbapenem. Upon reporting carbapenem resistance in the baseline pathogens by the local laboratory, physicians were permitted to discontinue the blinded treatment if patients did not respond to treatment [72, 79]. De-escalation of therapy was not feasible in the randomized APEKS–cUTI and APEKS–NP studies as patients received monotherapy [72, 79]. In the randomized CREDIBLE–CR study, de-escalation of adjunctive therapy was permitted based on local susceptibility reports; escalation of therapy after the early assessment visit was not permitted and administration of any other agent would be considered as “rescue therapy” for a patient without clinical improvement [73].

### APEKS–cUTI

The randomized APEKS–cUTI study was designed to demonstrate the non-inferiority of cefiderocol (2 g, q8h, infused over 1 h) to imipenem–cilastatin (1 g/1 g, q8h, infused over 1 h) in hospitalized patients with complicated urinary tract infection [79]. The enrolled patient population was at risk of being infected by MDR Gram-negative bacteria, including *P. aeruginosa*. Most patients were aged > 65 years, > 50% of patients had moderate or severe renal impairment, and the study design allowed the enrollment of patients with immunosuppression and renal transplant [79]. The study results showed that cefiderocol treatment was non-inferior to imipenem–cilastatin treatment in the primary endpoint of composite of microbiological eradication and clinical cure at the test-of-cure visit (Additional file 1: Table S1) [79]. Serious adverse events were reported in 5% and 8% of patients in the cefiderocol and imipenem–cilastatin arms, respectively [79].

### APEKS–NP

The randomized APEKS–NP study was conducted to demonstrate the non-inferiority of treatment with cefiderocol (2 g, q8h, 3-h infusion, 7–14 days) to high-dose, extended-infusion meropenem (2 g, q8h, 3-h infusion, 7–14 days) in day 14 all-cause mortality (ACM) rates among patients with Gram-negative NP [72]. A large proportion of the 292 patients included were critically ill, as shown by ICU admission (cefiderocol 70%, meropenem 66%), mechanical ventilation (cefiderocol 61%, meropenem 59%), Acute Physiology and Chronic Health Evaluation (APACHE) II scores ≥ 20 (cefiderocol 28%, meropenem 31%), age ≥ 75 years (cefiderocol 28%, meropenem 30%), and CrCl ≤ 50 mL/min (cefiderocol 33%, meropenem 35%) [72].

The study met its primary endpoint, with day 14 ACM rates of 12.4% with cefiderocol and 11.6% with meropenem (adjusted treatment difference 0.8%, 95% confidence interval (CI) –6.6 to 8.2) (Additional file 1: Table S1). In subgroup analyses, day 14 ACM rates were statistically comparable between treatments for patients with VAP, ventilated HAP, high APACHE II score and high Clinical Pulmonary Infection Score (CPIS), and ICU at randomization [72]. Comparable intertreatment-arm ACM rates were also seen at day 28, both overall (cefiderocol 21%, meropenem 21%) and for the most severely ill patients [72]. Furthermore, similar clinical cure and microbiological eradication rates were found between cefiderocol and meropenem arms by baseline Gram-negative pathogen (i.e., *K. pneumoniae*, *P. aeruginosa*, *A. baumannii* [including a proportion of meropenem-resistant isolates] and *E. coli*) [72].

### CREDIBLE–CR

The randomized, open-label, pathogen-focused, descriptive, Phase 3 CREDIBLE–CR study investigated the efficacy of cefiderocol and best available therapy (BAT) in 150 patients with serious infections (NP, BSI/sepsis, or cUTI) caused by CR pathogens, including non-fermenters, such as CR *P. aeruginosa* or CR *A. baumannii* [73]. Being designed with relatively few exclusion criteria, the CREDIBLE–CR study was able to enroll patients with underlying conditions who would normally be excluded from randomized, non-inferiority, double-blind Phase 3 studies, such as the APEKS–NP study. The heterogeneous nature of a population comprising different infection types necessitated the use of primary endpoints defined by infection type [73, 80]. The critically ill status of the population was reflected in the rates of ICU admission at randomization (cefiderocol 56%, BAT 43%), mechanical ventilation (cefiderocol 50%, BAT 53%), APACHE II scores ≥ 20 (cefiderocol 29%, BAT 27%), age ≥ 75 years (cefiderocol 29%, BAT 29%), CrCl ≤ 50 mL/min (cefiderocol 43%, BAT 30%), and shock (cefiderocol 19%, BAT 12%) [73]. There was a similarity between the cefiderocol and BAT treatment arms at randomization in CPIS scores among ventilated patients, Sequential Organ Failure Assessment (SOFA) scores, and Charlson Comorbidity Index [73].

Clinical cure rates, as primary endpoint, at the test-of-cure visit were similar for cefiderocol and BAT in patients with NP (50% and 53%, respectively) and BSI/sepsis (43% in each arm) [73]. The primary endpoint of microbiological eradication rate for baseline pathogen from urine for patients with cUTI was numerically higher in patients treated with cefiderocol (cefiderocol 53%, BAT 20%) [73]. Despite similar rates of clinical and microbiological outcomes between treatment arms, overall mortality rates were numerically higher in the cefiderocol arm than in the BAT arm by day 28 (25% and 18%, respectively) and end of study (34% and 18%, respectively) (Additional file 1: Table S1) [73].

### Sensitivity and subgroup analyses of the Phase 3 studies

Due to the higher observed ACM with cefiderocol relative to BAT in the CREDIBLE–CR study, several post-hoc analyses were performed. No single specific mortality risk factor was identified as a significant covariate on logistic regression modeling for the overall population [73]. The higher mortality rate in the cefiderocol arm was most apparent among patients with *A. baumannii* infections (cefiderocol 38%, BAT 18% at day 28; cefiderocol 50%, BAT 18% at end of study) [73]. Among patients without *Acinetobacter* spp. at randomization, mortality rates were similar between the cefiderocol and BAT arms at all visits (cefiderocol 15%, BAT 19% at day 28; cefiderocol 22%, BAT 19% at end of study) [73]. It is possible that imbalances in baseline factors within the heterogeneous population, together with limited stratification factors, contributed to the mortality differences between treatment arms [73, 81, 82]. The numerical differences between the treatment arms in ICU admission (cefiderocol 81%, BAT 47%), age ≥ 65 years (cefiderocol 62%, BAT 41%), Charlson Comorbidity Index ≥ 6 (cefiderocol 50%, BAT 35%), moderate or severe renal impairment (cefiderocol 33%, BAT 18%), and ongoing or prior shock (cefiderocol 26%, BAT 6%), point to the inclusion of more severely ill patients with CR *Acinetobacter* spp. in the cefiderocol arm compared with the BAT arm [73]. Most patients in the cefiderocol arm received monotherapy, while most patients in the BAT arm were treated with a combination of two or three antibiotics [73]. It is notable that the increased mortality rate in the cefiderocol arm was not linked to toxicity; the composite endpoint of survival and no change in antibiotic due to lack of therapeutic efficacy or emerging toxicity was reported in a similar proportion of patients in both treatment arms [73]. Furthermore, the observed mortality rate in the BAT arm for patients with CR *A. baumannii* infections was relatively low compared with rates in the control arms of previous randomized, controlled studies [82]. A post-hoc analysis of pneumonia patients infected by *Acinetobacter* spp. with meropenem MIC > 8 µg/mL in the double-blind, randomized APEKS–NP study showed comparable mortality rates at days 14 (cefiderocol: 28%, meropenem: 28%) and 28 (cefiderocol: 33%, meropenem: 39%) between cefiderocol and meropenem arms, respectively [72].

Based on the findings from the CREDIBLE–CR study, a “Warning and Precaution” (i.e., “Increase in all-cause mortality in patients with carbapenem-resistant Gram-negative bacterial infections: an increase in all-cause mortality was observed in cefiderocol-treated patients compared to those treated with best available therapy [BAT]) was added to the US prescribing information for cefiderocol. Although the cause of the increase in mortality has not been established, clinicians are advised to “closely monitor the clinical response to therapy in patients with cUTI and HABP/VABP" [49]. In addition, a special warning was included in the European Summary of Product Characteristics [51].

A subgroup analysis of the randomized APEKS–NP and CREDIBLE–CR studies confirmed the efficacy of cefiderocol in infections caused by CR Gram-negative pathogens harboring MBL enzymes (i.e., clinical cure: 70.8% and microbiological eradication: 58.3%), and no increased mortality was observed in these infections [83]. Among patients treated with comparators, clinical cure (40.0%) and microbiological eradication (30.0%) rates were lower than with cefiderocol [83]. The clinical benefit of cefiderocol treatment was consistent between the APEKS–NP and the CREDIBLE–CR studies [83]. *K. pneumoniae* harboring OXA-48 oxacillinase enzyme was detected across the CREDIBLE–CR and APEKS–NP studies in ten patients with cUTI, BSI/sepsis or pneumonia treated with cefiderocol [84]. All ten patients survived by day 28 and clinical cure at test of cure was reported for seven patients [84].

Given the role of iron channels in mediating cefiderocol entry into bacterial cells, the impact of patient iron levels on the clinical activity of cefiderocol merits attention. Anemia is not uncommon among critically ill patients and was reported for 8% of patients in each of the Phase 3 studies (see Safety section, below). A subgroup analysis of the APEKS–NP study showed that cefiderocol efficacy in critically ill patients with NP was not compromised by low serum iron levels at randomization [85] or by administration of iron supplementation in the form of blood transfusion or iron medications [85]. Of note, the majority of patients (~ 80%) had low baseline iron levels and this was most frequent among patients with ICU admission at randomization (71.9%) and those who were ventilated (63.2%) [85].

A post-hoc analysis of 84 patients with secondary bacteremia from the APEKS–cUTI, APEKS–NP, CREDIBLE–CR studies suggested that cefiderocol treatment may be an effective treatment in eradication of blood isolates of both carbapenem-susceptible and carbapenem-resistant Gram-negative species with low rates of persistence or recurrence [86].

### Safety

As would be expected in a critically ill patient population, rates of treatment-emergent adverse events (TEAEs) were relatively high in both treatment arms in the randomized APEKS–NP and CREDIBLE–CR studies. In the APEKS–NP study, the most common TEAE occurring with cefiderocol among 148 patients in the safety population was cUTI (15.5%), followed by hypokalemia (10.8%), diarrhea (8.8%), anemia (8.1%), pneumonia (7.4%), aspartate aminotransferase increase and pleural effusion (6.8%, each), alanine aminotransferase increase (6.1%), and hypomagnesemia (5.4%). Discontinuation due to drug-related adverse events was rare in both treatment arms [72]. In the CREDIBLE–CR study, nearly all patients had at least one TEAE [73]. The most common TEAEs occurring among 101 patients receiving cefiderocol in the safety population were diarrhea (19%), pyrexia (14%), septic shock and vomiting (13% each), decubitus ulcer (10%), and hypokalemia (9%), with abnormal liver function test, constipation, hypotension, anemia, aspartate aminotransferase increase, and pleural effusion each being reported in 8% of patients, acute kidney injury, dyspnea, nausea, pneumonia, and alanine aminotransferase increase each occurring in 7% of patients, and abdominal pain, hypomagnesemia, thrombocytopenia, and chest pain each being reported in 6% of patients [73]. Three patients (3%) receiving cefiderocol and two patients (4%) receiving BAT discontinued study drug due to drug-related TEAEs [73]. Drug-related serious adverse events occurred in one patient (1%) in the cefiderocol arm and five patients (10%) in the BAT arm [73]. In the cefiderocol arm, 30% of patients had liver-related TEAEs, but many of these patients had confounding factors (e.g., hepatitis) [73].

Across all three clinical studies, seizures were reported in one patient receiving cefiderocol (with a history of epilepsy) in the APEKS–cUTI study, three events (one event of which was deemed a serious adverse event [SAE]) in the cefiderocol arm and two events in the meropenem arm in the APEKS–NP study, and one mild event in the cefiderocol arm and one SAE in the BAT arm in the CREDIBLE–CR study [87]. *Clostridioides difficile* infection was observed in similar proportions of patients in the cefiderocol and comparator treatment arms in the APEKS–NP study (four patients [3%] in both the cefiderocol and meropenem arms, respectively) [72], in the CREDIBLE–CR study (three patients [3%] in the cefiderocol arm and one patient [2%] in the BAT arm) [87], and in one patient (< 1%) in the cefiderocol arm and five patients (3%) in the imipenem–cilastatin arm of the APEKS–cUTI study [87]. *C. difficile* SAEs were reported only in the APEKS–cUTI study, in one patient receiving cefiderocol and in two patients receiving imipenem–cilastatin [87].

As well as looking at the role of iron-related parameters in terms of efficacy, as discussed earlier, consideration has been given to their impact on safety. In the CREDIBLE–CR study, 78.4% of the 134 patients randomized who had baseline serum iron data available had low iron levels (cefiderocol 76.2%, BAT 57.1%) [88]. Iron levels did not appear to influence safety. Day 28 ACM rates were similar in the subset of patients who received either blood transfusion or iron supplementation during antibiotic treatment (cefiderocol 23.8%, BAT 27.8%) and did not increase among patients without any supplementation (cefiderocol 25.4%, BAT 12.9%) [89]. In all three randomized clinical trials, safety parameters related to iron homeostasis (i.e., hepcidin, iron, total iron-binding capacity, transferrin saturation) were similar between treatment arms [72, 73, 85, 87, 89].

## Real-world evidence

Several cases have been reported in which critically ill patients, often complicated with end-stage renal disease requiring CRRT, respiratory failure requiring ECMO, transplantation, hematological or solid cancer, other forms of immunosuppression, presence of coronavirus disease 2019 (COVID-19), burn wounds, or concurrent bacteremia, were successfully treated with cefiderocol either as monotherapy or in combination treatment (Table 4). Most patients had CR or extensively drug resistant (XDR) *A. baumannii*, CR or MDR *P. aeruginosa*, with CR *K. pneumoniae* and/or *S. maltophilia* co-infections, and clinical improvement or resolution was reported for the majority of patients. The mortality rates in case series reports (i.e., 23–55%) (Table 4) were similar to those observed in clinical studies [53, 58, 90–115].

**Table 4:** Summary of real-world evidence with cefiderocol treatment in critically ill patients

Investigation	No. of patients, sex, age (yr)	Species	Cefiderocol (monotherapy or combination therapy)	Comparator	Infection type	Outcome	Other parameters	References
1	*N* = 124; 92 M, median age ~ 65.5 yrs	CR AB	47 cases (15 monotherapy)	Colistin, 77 cases	BSI 79, VAP 35, other 10	Day 30 ACM 34% versus 55.8%, *P* = 0.018 8 pts had micro failure with cefiderocol, and 4 pts had resistance	Nephrotoxicity in colistin group, 90% in the ICU, 40% had COVID-19, higher % on ECMO in cefiderocol group during sepsis, similar % had CRRT during sepsis, 8 pts had solid cancer	[90]
2	*N* = 13; 13 M, mean age 61 yrs	CR AB	13 cases (8 monotherapy)	None	All NP	30-day clinical success 54% 30-day mortality 46%	12 pts polymicrobial, 12 pts had COVID-19, 11 pts in the ICU, 1 pt with immunosuppression	[92]
3	*N* = 17; 14 M, 3 F, median age 64 yrs	XDR, DTR PA	17 cases (3 monotherapy)	None	VAP 7, IAI 4, BSI 1, meningitis 1, empyema 1, NP 1, osteomyelitis 1, ecthyma gangrenosum 1	Clinical cure 70.6%, Clinical relapse in 3 pts, Eradication 76.5%, Day 30 ACM 23.5%	Pts had prior treatment failure, 88% in ICU; 3 pts had polymicrobial infection (1 PDR AB, 1 SM, 1 CR, CZA-R KP), 5 pts with COVID-19, 7 pts with immunosuppression	[93]
4	*N* = 107; 82 M, 25 F, median age 65 yrs	CR AB	42 cases (42 monotherapy)	Colistin, 65 cases, 82% combination therapy	BSI 58%, LRTI 41%	Day 28 ACM: Cefiderocol 55% Colistin 58% Clinical cure: Cefiderocol 40% Colistin 36%	Severe COVID-19, 9 pts with immunosuppression	[91]
5	*N* = 13; 8 M, 5 F, mean age 62.2 yrs	XDR AB	13 cases (10 monotherapy)	None	BSI + VAP 6, VAP 5, BSI 2	Microbiological failure 54%, Microbiological failure with suboptimal *f*C_min_/MIC 80%, Day 30 mortality 31%	All pts had COVID-19 pneumonia, TDM-driven assessment, 4 pts with ECMO, 2 pts with CRRT	[94]
6	*N* = 1; M, 64 yrs	PA	1 case (1 combination therapy; colistin + fosfomycin)	None	Empyema, pneumonia	Eradication	COVID-19, thoracostomy drainage, surgical debridement	[95]
7	*N* = 1; F, 55 yrs	PDR AB	1 case (1 combination therapy; sulbactam–durlobactam)	None	VAP	Clinical status improved and discharged	COVID-19, respiratory failure, mechanical ventilation, vasopressors, thoracostomy tube	[96]
8	*N* = 1; F, 64 yrs	MDR PA	1 case (1 combination therapy; polymyxin B + tobramycin)	None	Pneumonia, IAI (abscesses), BSI	Clinical improvement	ESRD, ESLivD, combined liver + kidney transplant, ARDS, ECMO, vasopressor and acute tubular necrosis requiring CVVHDF Polymicrobial infection	[97]
9	*N* = 13; NA, 21–72 yrs	CR PA, AB, KP, *E. hormaechei*	13 cases (4 monotherapy)	None	RTI 10, IAI 2, osteo-articular 2, SSSI 1, UTI 1	Clinical cure 54%, Clinical cure was observed among patients with cefiderocol-susceptible isolates, Mortality 23%	10 pts in the ICU, 7 pts with immunosuppression	[100]
10	*N* = 1; M, 63 yrs	XDR PA	1 case (1 monotherapy + metronidazole)	None	IAI, BSI	Favorable clinical evolution, and later died due to ischial eschar complication following treatment	Septic shock, diabetic foot ulcer	[101]
11	*N* = 2; M, 60 yrs, F, 70 yrs	Case 1 CR KP, PDR AB and Case 2 XDR AB, XDR PA	2 cases (2 monotherapy)	None	BSI	Case 1: clinical improvement and clearance of XDR AB Case 2: clinical improvement and clearance of XDR AB, XDR PA Both patients died from additional complications	2 polymicrobial cases, CRRT in both patients, Septic thrombosis post cardiothoracic surgery + myelotoxicity Persistent bacteremia	[102]
12	*N* = 10; F 60%, M 40%, Median 75.5 yrs	CR AB, SM, NDM-producing KP	10 cases (9 monotherapy, 1 combination therapy; fosfomycin)	None	BSI 60%, VAP 40%	Clinical success 70%, Day 30 mortality 10%, 2 cases of recurrent AB BSI	10 pts had mechanical ventilation, 2 pts with CRRT 5 pts had COVID-19, 4 pts had burns, 1 pt had colon perforation, 1 pt with cancer	[103]
13	*N* = 1; F, 10 yrs	PDR *Achromobacter*	1 case (1 combination therapy; meropenem–vaborbactam + bacteriophage)	None	RTI, acute pulmonary exacerbations	Eradication, improvement in respiratory function	CF with respiratory failure, declining respiratory function	[104]
14	*N* = 1; F, 45 yrs	XDR PA	1 case (1 monotherapy)	None	Empyema (esophageal–pleural fistula, pneumothorax)	Clinical improvement, but resistance emerged during treatment with cefiderocol	Complicated patient, cranial surgery	[105]
15	*N* = 1; M, 62 yrs	XDR AB	1 case (1 combination therapy; colistin + daptomycin + fluconazole)	None	Empyema	Complete resolution of fever, radiological signs, pleural cavity, urine culture	Invasive ventilation, hemothorax, surgery, AKI, polymicrobial infection	[106]
16	*N* = 1; M, 46 yrs	MDR PA	1 case (1 monotherapy + metronidazole)	None	IAI (abscess)	Clinical resolution	Hemodialysis, ESRD, polymicrobial infection	[107
17	*N* = 1; F, 68 yrs	CR KP	1 case (1 combination therapy; ceftazidime–avibactam, polymyxin B)	None	BSI, IAI	Eradication from blood, clinically stabilized, but fatal due to subsequent condition	Kidney transplant, ESRD, hemodialysis, polymicrobial infection	[108]
18	*N* = 1; M, adult	XDR AB, KPC KP	1 case (1 monotherapy + linezolid)	None	VAP, BSI	Complete resolution of fever, lung infiltrates	ECMO for respiratory failure due to viral pneumonia, treatment failure on prior colistin + AKI	[109]
19	*N* = 1; F, 78 yrs	ESBL, XDR PA + rectal colonization OXA-48 KP + OXA-23/OXA-51 AB	1 case (1 combination therapy; meropenem + colistin)	None	BSI, endocarditis	Eradication	Intermittent hemofiltration, sepsis, travel history	[110]
20	*N* = 1; M, 65 yrs	*E. coli*, XDR AB	1 case (1 combination therapy; ampicillin–sulbactam + tigecycline)	None	VAP	Microbiological cure with recurrence	CVVHDF, COPD, thoraco-abdominal aortic aneurysm surgical repair	[98]
21	*N* = 24; 17 M, 7 F, median age 66.5 yrs	AB 58%, PA 42%, KP 17%, SM 8%	24 cases (16 monotherapy, 8 combination therapy; minocycline, gentamycin, colistin, tobramycin)	None	VAP 79%, wound infection 17%, cUTI 4%, driveline infection 4%, concurrent BSI 21%	Clinical success 46%, clinical failure 54%, Day 30 ACM 42%	5 pts had COVID-19 pneumonia, mechanical ventilation and bacterial superinfection, 8 pts received hemodialysis	[99]
22	*N* = 13; 11 M, 2 F, median age 63 yrs	10 CR AB, 1 KPC-KP, 2 XDR PA	13 cases (13 combination therapy; colistin, fosfomycin, tigecycline, meropenem)	None	BSI 8, VAP/ pneumonia 3, abscess 2, neurosurgical wound infection 1, perihepatic abscess 1	All patients had clinical or microbiological cure, Day 30 ACM 23%	7 pts had history of COVID-19, 4 pts with post-surgical infection, 4 pts with immunosuppression	[111]
23	*N* = 5; 4 M, 1 F, age 35–76 yrs	MDR & CR AB, MDR PA, CFDC-R PA	5 cases (5 monotherapy)	None	CAP 20%, HAP 60%, sepsis 20%	Microbiological cure 60%, clinical cure 20%, death 40%	3 pts had CVVHD, 2 pts had ECMO, 2 pts had COVID-19, 2 pts were immunocompromised, 1 PA isolate was resistant to cefiderocol prior to treatment	[53]
24	*N* = 10; 9 M, 1 F, age 33–78 yrs	XDR/DTR PA, MDR PA, MDR *E. coli*, MDR *A. xylosoxidans*	10 cases (3 monotherapy, 7 combination therapy)	None	Bacteremia 40%, VAP 40%, other 20%	Clinical cure 90%, microbiological cure 80%, recurrence 10%, Day 30 ACM 10%	All patients were treated with CFDC as last resort, 5 pts had sepsis, 2 pts had septic shock, 1 pt had CVVHDF, 4 pts with immunosuppression	[112]
25	*N* = 1; M, 64 yrs	CR PA, SM	1 case (1 monotherapy)	None	RTI	Clinical cure, microbiological eradication, survival	SARS-CoV-2 infection, respiratory failure, ICU admission, venovenous ECMO	[58]
26	*N* = 3; 3 M, age 45–71 yrs	3 DTR AB, plus *E. faecalis* and *E. faecium* 1 pt, KP 1 pt	3 cases (1 monotherapy, 2 combination therapy; fosfomycin, nebulized colistin)	None	VAP 100%, sepsis 33.3%, BSI 66.7%	Clinical improvement 100%, microbiological eradication 100%, new infection 1 pt, death 1 pt	All 3 pts had acute renal injury requiring CVVH, mechanical ventilation, septic shock, vasopressor support 1 pt had bilateral lung transplant	[113]
27	*N* = 1; M, 57 yrs	*Achromobacter xylosoxidans*	1 case (1 combination therapy; fosfomycin, trimethoprim–sulfamethoxazole)	None	Endocarditis, sepsis	Clinical cure	SARS-CoV2 infection, ARDS Stage IVB Non-Hodgkin lymphoma	[114]
28	*N* = 10; 8 M, 2 F, age 30–85 yrs	6 PA, 1 AB, 1 SM, 1 *Burkholderia cenocepacia*, 1 *P. putida* complex	10 cases (4 monotherapy, 6 combination therapy; colistin, amikacin)	None	VAP 40%, UTI 20%, cholangitis 10%, otitis media 10%, central catheter infection 20%, secondary BSI 40%	Clinical response 20%, Microbiological failure 60%, Day 30 mortality 60%	6 pts with septic shock, 8 pts with immunosuppression	[115]

*AB A. baumannii*, *ACM* all-cause mortality, *AKI* acute kidney injury, *ARDS* acute respiratory distress syndrome, *BSI* bloodstream infection, *CAP* community-acquired pneumonia, *CF* cystic fibrosis, *CFDC* cefiderocol, *COPD* chronic obstructive pulmonary disease, *COVID-19* coronavirus disease 2019, *CR* carbapenem resistant, *CRRT* continuous renal replacement therapy, *CVVH* continuous venovenous hemofiltration, *CVVHDF* continuous venovenous hemodiafiltration, *cUTI* complicated urinary tract infection, *CZA-R* ceftazidime–avibactam*-*resistant, *DTR* difficult-to-treat resistance, *ECMO* extracorporeal membrane oxygenation, *ESBL* extended-spectrum beta-lactamase, *ESLivD* end-stage liver disease, *ESRD* end-stage renal disease, *F* female, *fC*_*min*_*/MIC* ratio of the minimum free-drug concentration of cefiderocol over the minimum inhibitory concentration of the pathogen, *IAI* intra-abdominal infection, *ICU* intensive care unit, *KP K. pneumoniae, KPC Klebsiella pneumoniae*-carbapenemase, *LRTI* lower respiratory tract infection, *M* male, *MDR* multidrug resistant, *MIC* minimum inhibitory concentration, *NDM* New Delhi metallo-beta-lactamase, *NP* nosocomial pneumonia, *OXA* oxacillinase, *PA P. aeruginosa*, *PDR* pan-drug resistant, *Pts* patients, *R* resistant, *RTI* respiratory tract infection, *SARS-CoV-2* Severe Acute Respiratory Syndrome coronavirus-2, *SM S. maltophilia*, *SSSI* skin and skin structure infection, *TDM* therapeutic drug monitoring, *UTI* urinary tract infection, *VAP* ventilator-associated pneumonia, *XDR* extensively drug resistant, *yrs* year

The two largest real-world, observational, retrospective studies involved patients with CR *A. baumannii* infections, who received either cefiderocol or colistin-containing regimens [90, 91] (Table 4). In one of these two studies, 124 patients were diagnosed mainly with BSI (63.7%) and VAP (28.2%), approximately 90% of patients were in the ICU and 40% had COVID-19 pneumonia, and a small percentage required ECMO (~ 15% among cefiderocol-treated patients) or CRRT (~ 12%) during sepsis. Patients receiving cefiderocol had a significantly lower 30-day mortality rate than those receiving colistin therapy (34% versus 55.8%, respectively, *p* = 0.018). The lower mortality rate with cefiderocol treatment at days 14 and 28 was still significant for patients with BSI, but not for patients with VAP [90]. A multivariate analysis showed that septic shock, higher SOFA score, and age were linked with increased risk, and cefiderocol-containing treatment with lower risk of mortality [90]. The second study included 107 critically ill patients in the ICU with COVID-19 pneumonia requiring mechanical ventilation, who were colonized by CR *A. baumannii* prior to infection, and had high median SOFA score (cefiderocol 9, colistin-based 8). Patients had LRTI (41%) or BSI (58%). The clinical and microbiological cure rates were similar at day 14 in cefiderocol-treated patients and those receiving other antibiotics [91]. Despite clinical improvement by day 14 as indicated by improvements in SOFA score in both treatment groups, the 14-day and 28-day mortality rates were similar with cefiderocol monotherapy (40% and 55%, respectively) and colistin-based therapy (51% and 58%, respectively) [91].

Further real-world evidence on cefiderocol use and outcomes is provided by the ongoing PROVE retrospective chart review study in US and European hospitals [116, 117]. Among 76 patients with *A. baumannii* infections recorded as receiving cefiderocol, 18% had at least four major comorbid conditions, 13% had COVID-19 infection during index hospitalization, 54% were in the ICU, and 41% were receiving mechanical ventilation [116]. Altogether, 36% of infections were polymicrobial, with *P. aeruginosa* as the main accompanying pathogen (33%). Most (96%) *A. baumannii* isolates tested were CR. Cefiderocol was administered as monotherapy in 55% of patients and following failure on another Gram-negative antibiotic in 11% of patients. The overall clinical cure rate was 63% and the 30-day ACM rate was 21%; among the 76% of patients receiving cefiderocol within the first week of positive culture, the cure rate was 64% and the 30-day ACM rate was 21% [116]. An overall clinical cure rate of 63% and a 30-day ACM rate of 21% were also reported among 120 patients receiving cefiderocol for *P. aeruginosa* infections caused mainly by CR isolates (97% of 114 isolates tested) [117]. Among 85 (71%) patients receiving cefiderocol within a week of positive culture, the clinical cure and 30-day ACM rates were 65% and 21%, respectively. Of the total cohort of patients with *P. aeruginosa* infections, one-quarter of patients (23%) had at least four major comorbid conditions, 14% had COVID-19 infection during index hospitalization, 76% were in the ICU, and 55% were receiving mechanical ventilation. Polymicrobial infections were recorded in 36% of cases, mainly with *A. baumannii* (23%). Cefiderocol was administered mostly as monotherapy (68%) and 12% of patients received it following failure on another Gram-negative antibiotic [117].

Viale et al. conducted a systematic literature review involving 150 cefiderocol-treated patients in the real-world setting from 44 studies/reports [118]. The most frequent clinical diagnosis was pneumonia (22%), and *P. aeruginosa* and *A. baumannii* were the two most frequent pathogens [118]. The duration of cefiderocol treatment ranged between 2 weeks and > 6 weeks, although it was not reported for 35% of patients. Across all studies, the overall clinical response, microbiological cure, and mortality rates were 80%, 72%, and 35%, respectively [118].

A case series of patients with VAP and/or BSI caused by *A. baumannii* aimed to find an association between cefiderocol C_min_/MIC ratio and microbiological outcomes. In this investigation, four patients received ECMO support (one also received CRRT) and had either optimal or quasi-optimal *C*_min_/MIC ratios. ECMO support did not appear to have an influence on the microbiological result following cefiderocol treatment [94]. In a recent report, one patient without comorbid conditions, who developed VAP and sepsis, received ECMO and was infected by XDR/DTR *A. baumannii*, New Delhi metallo-beta-lactamase (NDM)-producing *K. pneumoniae*, and *Candida auris*. Following cefiderocol treatment with dose adjustment, clinical cure, microbiological cure was reported for the patient with survival at day 30 [112].

## Resistance emergence on-therapy

Although clinical cases of resistance emerging during therapy have been reported [90, 101, 105, 119–121], resistance to cefiderocol remains relatively uncommon. It is likely that on-therapy resistance requires a variety of concurrently present mechanisms, such as increased expression of specific MBLs (e.g., NDM), mutations in AmpC beta-lactamase, siderophore receptors, and regulators of iron transport channels, and/or target (penicillin-binding protein-3) modifications [122, 123]. Reports indicate that certain mutations in iron-transport genes, which are species specific, do not increase cefiderocol MIC values consistently above the susceptibility breakpoints [19], unless other resistance mechanisms are also present [123]. A cefiderocol-resistant NDM-producing *K. pneumoniae* strain obtained in vitro and due to functional loss of the iron transporter gene *cirA* was outcompeted by the parent strain in vitro, thus showing a fitness defect that suggests this resistance mechanism has low propensity to disseminate in the absence of strong selective pressure [124]. However, an outbreak of highly cefiderocol-resistant NDM-producing *K. pneumoniae* causing clinical infections, and due mainly to clonal expansion of a mutant with an inactivated *cirA* gene, has recently been observed in the absence of strong selective pressure [125].

Increases of ≥ four-fold in the MICs emerged in similar proportions of patients receiving cefiderocol and comparator antibiotics in both the randomized CREDIBLE–CR study (cefiderocol 15% [*A. baumannii*, *K. pneumoniae*, *P. aeruginosa* and *S. maltophilia*] and BAT 13% [*K. pneumoniae*, *A. baumannii*, *E. coli*]) and the randomized APEKS–NP study (cefiderocol 5% [*Enterobacter aerogenes*, *K. pneumoniae*, *E. cloacae* and *S. marcescens*] and meropenem 4% [*K. pneumoniae*, *P. aeruginosa* and *C. freundii*]), although in most cases the elevated cefiderocol MIC did not reach resistance [72, 73, 126]. To date in real-world cases, on-therapy resistance during or after cefiderocol treatment were reported for two patients with CR *P. aeruginosa* infections [101, 105], three patients with CRE infections [119, 120, 127], and five patients with CR or XDR *A. baumannii* infections [90, 121]. Regular monitoring of resistance emergence is needed for clinical use of cefiderocol.

## Role of cefiderocol in the ICU

Overall, published data indicate that cefiderocol is a useful addition to the antibiotic armamentarium against CRE, DTR-*P. aeruginosa*, CR *A. baumannii*, and rare species, such as *Achromobacter* spp., *S. maltophilia* or *Burkholderia* spp. [13, 14, 19, 23, 30, 72, 73]. Preclinical studies confirm that standard dosing leads to bactericidal activity of cefiderocol and population PK analyses show adequate exposure in plasma and ELF for the treatment of infections with cefiderocol MIC values up to 4 µg/mL across all renal function groups [71, 76]. Phase 3 clinical studies showed similar or statistically comparable efficacy in critically ill patients between cefiderocol and comparator antibiotics at standard and renal function-adjusted doses, including patients with ARC [72, 73]. In addition, clinical studies and real-world case reports in critically ill patients also confirm the similar safety of cefiderocol to other beta-lactams, and that an increase in ACM rates in the cefiderocol arm of the randomized CREDIBLE–CR study was not due to toxicity. In the randomized CREDIBLE–CR study, notable baseline imbalances were observed between the two treatment arms among patients with CR *A. baumannii* infections, making interpretation of efficacy and ACM findings difficult [73]. This is further complicated by the finding that ACM among patients in the BAT arm with CR *A. baumannii* infections was substantially lower relative to the rates observed with other treatments in previous randomized, controlled studies [82]. Further studies are required to clarify the role of cefiderocol treatment for the patient population in which it is intended to be used.

Comparative clinical data with cefiderocol and other newer beta-lactam antibiotics are scarce. Surveillance studies have demonstrated that a large proportion of Gram-negative isolates are susceptible to cefiderocol even if they are non-susceptible to the newer BL–BLI agents (Table 2) [28, 30]. Cross-resistance between cefiderocol and the new BL–BLIs has been observed to be rare. Pathogens are also likely to remain susceptible to cefiderocol when the isolates carry an MBL gene or porin mutations [19]. Furthermore, cefiderocol is the only beta-lactam antibiotic that has activity against most clinically relevant, problematic non-fermenter species such as *P. aeruginosa*, *A. baumannii* complex, *S. maltophilia*, *Achromobacter* spp., and *B. cepacia* complex frequently encountered in the ICU or in certain patient groups (e.g., colonization in patients with cystic fibrosis [CF]) [19, 28]. Against *P. aeruginosa* resistant or not susceptible to a range of common antipseudomonal antibiotics, cefiderocol MICs ranged between ≤ 0.06 and 4 μg/mL for 98.3% of all isolates, and susceptibility rates were 97.4% and 97.9% against XDR and MDR isolates, respectively [128]. Thus, cefiderocol has the broadest spectrum in terms of species and resistance mechanisms present in CR Gram-negative pathogens (Table 5) [13, 19, 28, 30].

**Table 5:** Overview of recently approved beta-lactam–beta-lactamase inhibitors and cefiderocol with activity against carbapenem-resistant Gram-negative bacilli

	Cefiderocol [49, 51]	Ceftazidime–avibactam [148, 149]	Ceftolozane–tazobactam [150, 151]	Imipenem–relebactam [152, 153]	Meropenem–vaborbactam [154, 155]
Antibacterial coverage	Enterobacterales *Acinetobacter* *Pseudomonas* *Stenotrophomonas* *Achromobacter* *Burkholderia*	Enterobacterales *Pseudomonas*	*Pseudomonas*	Enterobacterales *Pseudomonas* *Anaerobic Gram-negative and Gram-positive bacteria*	Enterobacterales
Coverage of beta-lactamase producers	Serine-carbapenemases (Class A and Class D [including CRAB producing class D carbapenemases), AmpC enzyme, certain extended-spectrum beta-lactamase, metallo-carbapenemases	Serine-carbapenemases (Class A and Class D), Amp C enzyme, certain extended-spectrum beta-lactamase	Amp C enzyme, certain extended-spectrum beta-lactamase	Serine-carbapenemases (Class A), Amp C enzyme, certain extended-spectrum beta-lactamase	Serine-carbapenemases (Class A), Amp C enzyme, certain extended-spectrum beta-lactamase
Dosing recommendations	Normal, mild, moderate, severe renal impairment, ARC	Normal, mild, moderate, severe renal impairment	Normal, mild, moderate, severe renal impairment	Normal, mild, moderate, severe renal impairment	Normal, mild, moderate, severe renal impairment
Drug–drug interaction	Monitoring may be required	Monitoring may be required	Monitoring may be required	Monitoring may be required	Monitoring may be required
Compatibility	Yes	Yes	Yes	Yes	Yes
Clinical efficacy and safety	Pneumonia, UTI, sepsis, bacteremia, and infections with limited treatment options	Pneumonia, UTI, IAI, bacteremia, and infections with limited treatment options	Pneumonia, UTI, IAI	Pneumonia, UTI, IAI, bacteremia, and infections with limited treatment options	Pneumonia, UTI, IAI, bacteremia, and infections with limited treatment options
Lung penetration	Yes	Yes	Yes	Yes	Yes
Considerations	ACM was numerically increased with cefiderocol in the CREDIBLE–CR study in pneumonia, BSI, sepsis caused by *Acinetobacter* spp. On-therapy resistance	Decreased clinical efficacy in adult patients with IAI and moderate renal impairment On-therapy resistance	Decreased clinical efficacy in adult patients with IAI and moderate renal impairment On-therapy resistance	Monitoring of liver function, particularly in patients with liver disease Alternative therapies for patients with ARC On-therapy resistance	On-therapy resistance

*ACM* all-cause mortality, *ARC* augmented renal clearance, *BSI* bloodstream infection, *CRAB* carbapenem-resistant *Acinetobacter baumannii*, *IAI* intra-abdominal infection, *UTI* urinary tract infection

Positioning within expert guidelines is another useful way to assess the relative merits of newer and established agents [129–134]. The European Society of Clinical Microbiology and Infectious Diseases (ESCMID) guidelines [129] reviewed all available clinical evidence for each antibiotic through PICO questions (i.e., patients, intervention, comparator, outcome). However, because current recommendations by ESCMID for or against newer antibiotics in the treatment of severe CRE infections, DTR *P. aeruginosa*, and CR *A. baumannii* infections are often supported by low-quality evidence, the role of some of the newer antibiotics remains unclear [129]. Gatti et al. have developed different treatment algorithms based on types of resistance mechanism and infection [132–134]. These Italian recommendations are aligned in general with the ESCMID guidelines in that the recommendations for cefiderocol are cautious and its use in critically ill patients should be targeted to CRE, CR *P. aeruginosa*, and CR *A. baumannii* infections with certain resistance mechanisms when in vitro activity is confirmed [132–134]. The two Infectious Diseases Society of America (IDSA) guidances are based on anatomical infection site, resistance mechanisms, and species [130, 131]. Cefiderocol is recommended as an alternative treatment option for patients with complicated UTIs caused by CRE, for infections outside the urinary tract caused by MBL-producing CRE, or as an alternative agent for CRE infections outside the urinary tract due to KPC-producers [130]. In UTIs caused by DTR *P. aeruginosa*, cefiderocol is one of the preferred treatment options, and for infections outside the urinary tract, cefiderocol is an alternative option [130]. For the treatment of CR *A. baumannii* and moderate/severe *S. maltophilia* infections, cefiderocol is recommended in combination with other agents [131].

Consistent with best antibiotic stewardship practices, cefiderocol use should be considered as an early targeted treatment option in patients in whom an XDR, DTR, CR, or MDR infection is highly suspected or documented given the deleterious outcomes associated with delayed receipt of effective therapy [99]. However, susceptibility testing even for cefiderocol is highly recommended to ensure treatment of susceptible pathogens [99, 100]. Among the testing methods that have been developed, currently, the BMD method with iron-depleted medium provides consistently accurate susceptibility results for cefiderocol [24, 26]. Patients with an increased likelihood of MDR Gram-negative bacterial infection include those with prior hospitalization, prior antibiotic use within 90 days, previous infection or colonization with a drug-resistant bacteria in the gut, urinary tract or respiratory tract, age > 70 years, diabetes mellitus, chronic obstructive pulmonary disease, or malignancy; poor infection control in hospital, travel to endemic areas, and high rate of local antimicrobial resistance are also contributing factors [9, 10, 135, 136]. For ICU-acquired pneumonia or VAP, individual patient factors such as septic shock at the time of VAP, acute respiratory distress syndrome or acute renal replacement therapy prior to VAP, and chronic liver disease are also linked with increased risk of MDR infections [9, 10, 136, 137]. Real-world data show that over 10% of critically ill patients in the ICU with *A. baumannii* and *P. aeruginosa* infections, in whom cefiderocol is expected to also be an appropriate antibiotic choice [28, 30], currently receive cefiderocol as a last-resort antibiotic [116, 117]. Because cefiderocol susceptibility testing may lengthen times to obtain MIC results, the initiation of therapy in some clinical settings should not be delayed given the high susceptibility rates among most Gram-negative pathogens, which could be followed by de-escalation of therapy. Further clinical experience is needed to explore whether cefiderocol treatment, administered to at-risk patients prior to treatment failure with other antibiotics, is associated with improved outcomes.

Like other antibiotics for patients with VAP, optimal duration of cefiderocol merits further investigation. In the randomized APEKS–NP and CREDIBLE–CR Phase 3 studies, patients with pneumonia and BSI/sepsis received cefiderocol treatment on average of 10–11 days [72, 73]. However, data are conflicting on the optimal duration of therapy for patients with VAP. According to the ATS/IDSA guidelines, a 7-day treatment is recommended for patients with HAP or VAP when improvement in signs and symptoms can be detected early. However, prolonged treatment should be considered for patients without clinical improvement or for those complicated with secondary bacteremia [138]. In a prospective controlled study in France, the non-inferiority of short-term (8 days) versus long-term (15 days) antibiotic treatment could not be established in patients with VAP caused by *P. aeruginosa*, and the study was stopped early due to slow enrolment. A tendency was seen towards a more frequent recurrence among patients receiving a short-term treatment, but no impact was found on 28-day mortality rate, length of ICU stay, and duration of mechanical ventilation [139]. In contrast, in a meta-analysis of randomized clinical studies comparing the outcomes after short-term and long-term antibiotic treatment courses in patients with VAP, and specifically in the subgroup of patients with non-fermenting Gram-negative bacteria, no significant differences in the rates of recurrence or relapse were found [140]. However, delay in appropriate antibiotics is known to impact mortality rates in such at-risk patient populations. A recent study has shown that improvement in identification of MDR Gram-negative pathogens with specific resistance profile through a streamlined microbiology workflow shortens the time to select appropriate antibiotics [141]. Such intervention, combined with shorter treatment duration, potentially can have a favorable impact on patient outcomes and may result in equal rates of overall clinical and microbiological response as longer treatment duration. However, after end of antibiotic treatment, monitoring of patients is warranted to detect any relapse or superinfection that would require additional courses of antibiotic treatment, particularly among patients with risk factors for drug-resistant pathogens [138].

## Future directions

Future studies are needed to better define the clinical role of cefiderocol for critically ill patients with highly resistant Gram-negative infections. One area concerns combination therapy. Despite limited randomized Phase 3 clinical trial data (with cefiderocol being delivered exclusively as monotherapy in APEKS–NP and mainly as monotherapy [70–80%] in CREDIBLE–CR [72, 73]), real-world evidence supports the use of cefiderocol both as monotherapy and in combination [53, 58, 90–117]. This needs to be further investigated in clinical trials and optimal combination partners should be determined. Previous work with the in vitro chemostat model and the in vivo murine infection models point to the use of avibactam, amikacin and ampicillin/sulbactam [66–69].

Accurate systems for easy and ready-to-use susceptibility testing of cefiderocol by diagnostic laboratories are needed for improving drug prescription practice for definitive chemotherapy of infections caused by Gram-negative pathogens with limited treatment options. While initial testing by disk diffusion followed by confirmation of uncertain results by reference BMD with iron-depleted medium can be an acceptable option, evaluations of novel commercial testing systems will eventually indicate reliable alternatives in the future.

The need for routine therapeutic drug monitoring (TDM) in the ICU is still being debated [142–145]. Data from more than 500 patients in the Phase 1–3 clinical studies and population PK modeling suggest that in general routine TDM is not essential, because the current cefiderocol dosing recommendations are likely to achieve the PD target predicted by preclinical animal studies with very high probability in all infection types [70, 71, 76]. However, certain patient groups, such as those requiring CRRT and/or ECMO, may benefit from routine TDM [145], particularly when beta-lactam antibiotics are used for their treatment [142, 143]. Currently, data from cefiderocol TDM studies for cefiderocol are sparse [94, 97] and the contribution of host factors to outcomes cannot be discounted [94]. Two ongoing Phase 1 clinical studies (clinicaltrials.gov NCT04995835 and NCT05373615) may clarify dosing needs for these specific patient populations.

The emergence of resistance in clinical practice is a consideration for all new antibiotics. While the current level of non-susceptibility to cefiderocol based on CLSI breakpoints is very low (i.e., CRE 1.8%, XDR *P. aeruginosa* 2.8%, CR *A. baumannii* 4.2%) [30], resistant isolates may develop and spread [121]. On-therapy resistance development should be monitored, especially for patients with recurrent infections or for non-responders. Rapid diagnostic testing to identify the causative pathogen and underlying carbapenem resistance mechanisms [8] should be more widely adopted and used together with risk factor assessment to ensure an appropriate targeted approach in patient populations for whom timing of appropriate antibiotic treatment is critical.

Future clinical trials are required to help refine the role of cefiderocol in the management of seriously ill patients with CR Gram-negative bacterial infections. For example, the ongoing phase 2 GAMECHANGER trial is investigating the efficacy of cefiderocol versus investigator-determined BAT (within 48 h of the index blood culture) for patients with healthcare-associated and hospital-acquired Gram-negative BSI [146]. The trial is due to be completed in the first quarter of 2023. Additional information will also be provided by ongoing PK studies in patients with CF and pulmonary exacerbation (NCT05314764), as well as critically ill patients requiring ECMO (NCT04995835) or CRRT (NCT05373615). Finally, it will be essential to continue to monitor the benefits of cefiderocol opposite newer antibiotics as they enter the clinical arena.

## Conclusions

Based on the reviewed clinical evidence, dosing recommendations, including for patients with severe renal impairment, CRRT or ARC, as well as PK/PD and safety profiles, cefiderocol can be regarded as one of the treatment options available for CR Gram-negative infections in critically ill patients in the ICU, either as monotherapy or in combination. Consideration should be given to the severity of illness and/or the potential for resistance emergence. Risk factors associated with mortality should be carefully considered when antibiotic treatment, including cefiderocol, is selected for patients in the ICU.

## Supplementary Information

**Additional file 1: Table 1. **Summary of randomized clinical trial evidence for cefiderocol in the APEKS–cUTI, APEKS–NP and CREDIBLE–CR studies.

## Data Availability

Not applicable.

## Abbreviations

ACM: All-cause mortality
APACHE: Acute Physiology and Chronic Health Evaluation
ARC: Augmented renal clearance
AST: Antimicrobial susceptibility testing
ATU: Area of technical uncertainty
AUC: Area under the concentration–time curve
BAT: Best available therapy
BL–BLI: Beta-lactam–beta-lactamase inhibitor
BMD: Broth microdilution
BSI: Bloodstream infection
CAP: Community-acquired pneumonia
CA-UTI: Catheter-associated complicated urinary tract infection
CF: Cystic fibrosis
CFU: Colony-forming unit
CI: Confidence interval
CLSI: Clinical and Laboratory Standards Institute
C_max_: Maximum serum concentration
C_min_: Minimum serum concentration
COVID-19: Coronavirus disease 2019
CPIS: Clinical Pulmonary Infection Score
CR: Carbapenem resistant
CrCl: Creatinine clearance
CRE: Carbapenem-resistant Enterobacterales
CRRT: Continuous renal replacement therapy
cUTI: Complicated urinary tract infection
DTR: Difficult-to-treat resistant
ECMO: Extracorporeal membrane oxygenation
eGFR: Estimated glomerular filtration rate
ELF: Epithelial lining fluid
ESBL: Extended-spectrum beta-lactamase
ESCMID: European Society of Clinical Microbiology and Infectious Diseases
EUCAST: European Committee on Antimicrobial Susceptibility Testing
FDA: US Food and Drug Administration
%*f*T_>MIC_: Percentage of time during the dosing period that the free drug concentration in the plasma exceeds the MIC
HABP: Hospital-acquired bacterial pneumonia
HAP: Hospital-acquired pneumonia
HCAP: Healthcare-associated pneumonia
ICU: Intensive care unit
IDSA: Infectious Diseases Society of America
IQR: Interquartile range
IV: Intravenous
LRTI: Lower respiratory tract infection
MBL: Metallo-beta-lactamase
MDR: Multidrug resistant
MIC: Minimum inhibitory concentration
NDM: New Delhi metallo-beta-lactamase
NP: Nosocomial pneumonia
OXA: Oxacillinase
PD: Pharmacodynamics
PDR: Pandrug resistant
PICO: Patients, Intervention, Comparator, Outcome
PK: Pharmacokinetic
PTA: Probability of target attainment
q8h: Every 8 h
SAE: Serious adverse event
SARS-CoV-2: Severe Acute Respiratory Syndrome coronavirus-2
SD: Standard deviation
SOFA: Sequential organ failure assessment
TDM: Therapeutic drug monitoring
TEAE: Treatment-emergent adverse events
TOC: Test of cure
UTI: Urinary tract infection
VABP: Ventilator-associated bacterial pneumonia
VAP: Ventilator-associated pneumonia
XDR: Extensively drug resistant
