# Supplementary material for: Treatment of critically ill patients with cefiderocol for infections caused by multidrug-resistant pathogens: review of the evidence
Source: Ann Intensive Care. 2023 Jun 15;13:52. doi: 10.1186/s13613-023-01146-5 (PMC10272070; doi:10.1186/s13613-023-01146-5)
Supplement: Supplementary file 1 — Additional file 1: Table 1. Summary of randomized clinical trial evidence for cefiderocol in the APEKS–cUTI, APEKS–NP and CREDIBLE–CR studies. [file 13613_2023_1146_MOESM1_ESM.docx]

Treatment of critically ill patients with cefiderocol for infections caused by multidrug-resistant pathogens: review of the evidence

Pierluigi Viale^1,2^, Christian E Sandrock^3^, Paula Ramirez^4^, Gian Maria Rossolini^5,6^ and Thomas P. Lodise^7^

**Table S1.** Summary of randomized clinical trial evidence for cefiderocol in the APEKS-cUTI, APEKS-NP and CREDIBLE-CR studies

| **APEKS-cUTI** (Phase II, double-blind, randomized 2:1, non-inferiority study) [79] | | | | Primary endpoint | | Other endpoints | | |  |
| --- | --- | --- | --- | --- | --- | --- | --- | --- | --- |
|  | Diagnosis | Dosing | Treatment duration, days | Clinical + microbiological response at TOC^a,b^ | | Clinical cure at TOC^a,b^ | Microbiological eradication at TOC^a,b^ | |  |
| Cefiderocol arm  N=252 | cUTI and/or acute pyelonephritis caused by Gram-negative bacteria, known carbapenem-resistant infections were excluded | 2 g q8h (1h IV infusion) for 7–14 days | Mean: 9.0 (SD 2.7)^b^ | 183/252 (73%) | | 226/252 (90%) | 184/252 (73%) | |  |
| Imipenem-cilastatin arm  N=119 |  | 1 g/ 1 g q8h (1h IV infusion) for 7–14 days | Mean: 9.0 (SD 2.6)^b^ | 65/119 (55%) | | 104/119 (87%) | 67/119 (56%) | |  |
|  |  |  |  | Adjusted treatment difference: 18.58% (95% CI 8.23; 28.92; *P*=0.0004) | | Treatment difference: 2.39% (95% CI –4.66; 9.44) | Treatment difference: 17.25% (95% CI 6.92; 27.58) | |  |
| **APEKS-NP** (Phase III, double-blind, randomized 1:1, non-inferiority study) [72] | | | | Primary endpoint | | Other endpoints | | |  |
|  | Diagnosis | Dosing | Treatment duration, days | 14-day ACM^b^ | | Clinical cure at TOC^a,b^ | Microbiological eradication at TOC^a,b^ | |  |
| Cefiderocol arm  N=145 | HAP, VAP, HCAP caused by Gram-negative bacteria, known carbapenem-resistant infections were excluded | 2 g q8h (3h IV infusion) for 7–14 days | Mean: 10.4 (SD 4.1)^c^ | 18/145 (12.4%) | | 94/145 (65%) | 59/124 (48%) | |  |
| Meropenem arm  N=147 |  | 2 g q8h (3h IV infusion) for 7–14 days | Mean: 10.1 (SD 4.0)^c^ | 17/146 (11.6%) | | 98/147 (67%) | 61/127 (48%) | |  |
|  |  |  |  | Adjusted treatment difference: 0.8% (95% CI –6.6; 8.2) | | Adjusted treatment difference: –2.0 (95% CI –12.5; 8.5) | Adjusted treatment difference: –1.4 (95% CI –13.5; 10.7) | |  |
| **CREDIBLE-CR** (Phase III, open-label, randomized 2:1, descriptive study) [73] | | | | Primary endpoint | | Other endpoints | | |  |
|  | Diagnosis | Dosing | Treatment duration, days^c^ | NP, BSI/Sepsis: Clinical cure at TOC^a,d^ | cUTI: Microbiological eradication at TOC^a,d^ | Day 28 ACM^c^ | | End of study ACM^c^ | |
| Cefiderocol arm  N=80 | HAP, VAP, HCAP, BSI/sepsis, cUTI caused by carbapenem-resistant Gram-negative bacteria | 2 g q8h (3h IV infusion) for 7–14 days | NP, BSI/Sepsis: median 11.0 (IQR 8.0–14.0)  cUTI: median 10.5 (IQR 8.0–15.0) | NP: 20/40 (50%)  BSI/sepsis: 10/23 (43%) | 9/17 (53%) | 25/101 (25%) | | 34/101 (34%) | |
| Best available therapy arm  N=38 |  | Dosed according to country label | NP, BSI/Sepsis: median 13.0 (IQR 10.0–15.0)  cUTI: median 6.5 (IQR 6.0–11.0) | NP: 10/19 (53%)  BSI/sepsis: 6/14 (43%) | 1/5 (20%) | 9/49 (18%) | | 9/49 (18%) | |

Adapted from references 72,73,79.

N, number of patients in the primary efficacy endpoint patient population.

^a^ TOC = 7 days (± 2 days) after end of antibiotic treatment.

^b^ Modified intention-to-treat population.

^c^ Safety population.

^d^ Carbapenem-resistant, microbiological intention-to-treat population.

*ACM* all-cause mortality, *BSI* bloodstream infection, *CI* confidence interval, *cUTI* complicated urinary tract infection, *HAP* hospital-acquired pneumonia, *HCAP* healthcare-associated pneumonia, *IQR* interquartile range, *IV* intravenous, *NP* nosocomial pneumonia, *SD* standard deviation, *TOC* test of cure, *VAP* ventilator-associated pneumonia.
